# Supplementary material for: Pomegranate Pomace Extract with Antioxidant, Anticancer, Antimicrobial, and Antiviral Activity Enhances the Quality of Strawberry-Yogurt Smoothie
Source: Bioengineering (Basel). 2022 Nov 28;9(12):735. doi: 10.3390/bioengineering9120735 (PMC9774345; doi:10.3390/bioengineering9120735)
Supplement: Supplementary file 1 [file bioengineering-09-00735-s001.zip › bioengineering-2002588-supplementary.pdf]

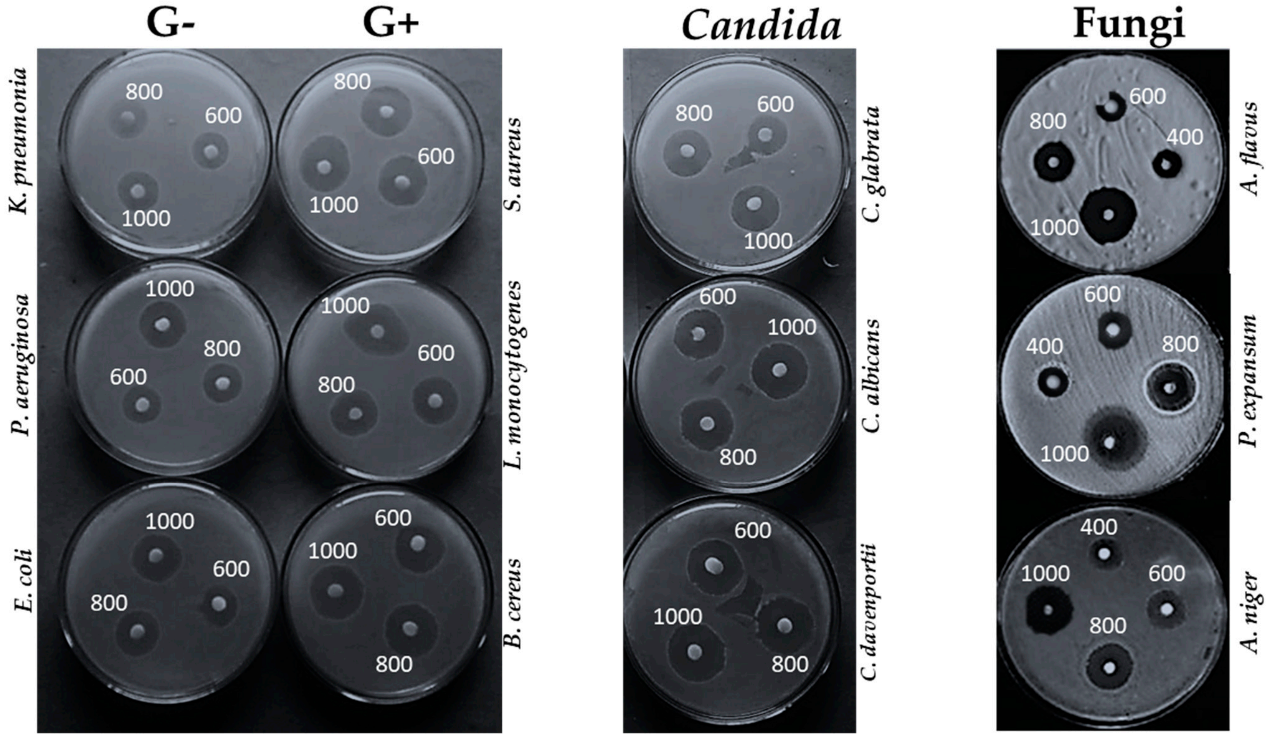

Figure S1. Inhibition zones diameters of PP extracts (0.6, 0.8, and 1.0 mg/mL) against tested bacteria (*S. aureus, L. monocytogenes, B. cereus, P. aeruginosa, K. pneumonia, E. coli*); Candida (*C. glabrata, C. albicans, C. davenportii*), and fungi (*A. niger, A. flavus, P. expansum*).
